# Metformin therapy and postoperative atrial fibrillation in diabetic patients after cardiac surgery

**DOI:** 10.1186/s40560-017-0254-8

**Published:** 2017-10-18

**Authors:** Suresh Basnet, Andrzej Kozikowski, Haiyan Sun, Melissa Troup, Luis E. Urrutia, Renee Pekmezaris

**Affiliations:** 1 Department of Critical Care Medicine, Valley Health System, Winchester, VA USA; 2 Department of Medicine, Northwell Health, Great Neck, NY USA; 3 Biostatistics Core, Geisinger Medical Center, Danville, PA USA; 4 Investigator-Initiated Research Operations, Geisinger Medical Center, Danville, PA USA; 5 Department of Critical Care Medicine, Geisinger Medical Cent, Danville, PA USA; 6 Department of Cardiovascular Medicine, Geisinger Medical Center, Danville, PA USA

**Keywords:** Cardiac surgery, Metformin, Postoperative atrial fibrillation, Diabetes

## Abstract

**Background:**

Postoperative atrial fibrillation (AF) commonly occurs in cardiac surgery patients. Studies suggest inflammation and oxidative stress contribute to postoperative AF development in this patient population. Metformin exerts an anti-inflammatory effect that reduces oxidative stress and thus may play a role in preventing postoperative AF.

**Methods:**

We conducted a matched, retrospective cohort study of diabetic patients’ age ≥18 undergoing a coronary artery bypass graft (CABG) and/or cardiac valve surgery from January 1, 2009, to November 30, 2014. We extracted data from The Society of Thoracic Surgeons National Adult Cardiac Surgery Database. Primary exposure was ongoing metformin use at a dose of ≥ 500 mg in effect before cardiac surgery as captured before admission. Primary study outcome was postoperative AF incidence. Matching was used to reduce selection bias between metformin and non-metformin groups. Comparison between the groups after matching was accomplished using the McNemar test or paired *t* test.

**Results:**

Out of the 4177 patients with cardiac surgery (CABG and/or valve surgery), 1283 patients met our study criteria. These patients were grouped into metformin [*n* = 635 (49.5%)] and non-metformin [*n* = 648 (50.5%)] users. Pre-matching, postoperative AF was found in 149 (23.5%) patients in the metformin group and 172 (26.5%) in the non-metformin group (*p* = 0.2088). Matching resulted in a total of 114 patients in each group (metformin vs. non-metformin). We found no statistically significant difference for postoperative AF between the two groups after matching (*p* = 0.8964).

**Conclusions:**

Prior use of metformin therapy in diabetic patients undergoing cardiac surgery was not associated with decreased rate of postoperative AF.

## Background

The incidence trend of postoperative atrial fibrillation (AF) has remained high over the last two decades, ranging from 10 to 65% depending on patient characteristics and preoperative as well as perioperative risk factors [1–4]. Postoperative AF is associated with greater resource utilization, prolonged hospital stays, increased morbidity, and in-hospital and long-term mortality [3–6].

The literature shows that beta blockers and anti-arrhythmic agents decrease the risk of developing postoperative AF [7]. Thus, the American Association of Thoracic Surgery and Canadian Cardiovascular Society both recommend the use of beta blockers and anti-arrhythmic agents such as amiodarone to prevent postoperative atrial fibrillation [8, 9]. However, as of today, there is no standard therapy to effectively avoid the development of this well-known postoperative cardiac surgery complication.

Metformin, an oral hypoglycemic agent, exerts an anti-inflammatory effect by reducing C-reactive protein (CRP) in both humans [10] and animal models [11]. Esteghamati et al. showed that metformin significantly reduced YKL-40, a novel marker of inflammation and oxidative stress in patients newly diagnosed with type 2 diabetes (T2D) [12, 13]. Metformin therapy has also been found by Chang et al. to be associated with decreased risk of AF in patients with T2D not using other anti-diabetic agents [14].

With the exception of a few studies, the relationship between metformin use and the development of AF has not been investigated. The main objective of this study was to investigate the role of metformin therapy in the prevention of postoperative AF in diabetic cardiac surgical patients.

## Methods

A retrospective cohort study was conducted utilizing data from The Society of Thoracic Surgeons (STS) Adult Cardiac Surgery Database Version 2.73. The STS National Database is one of the largest databases in medicine used in the analysis and reporting of risk-adjusted outcomes in cardiothoracic surgery [15]. The database contains over 5.9 million surgical records and collects data from approximately 90% of the US institutions that perform cardiac surgery [16]. A detailed description of the STS database design can be found elsewhere [17, 18]. The database was utilized to obtain a list of eligible patients. Cross-referencing from the electronic medical record was conducted to obtain the remaining data elements needed for inclusion criteria (e.g., medications). Geisinger Institutional Review Board (IRB#2014-0627) approval was obtained before study initiation.

Data extracted from the STS database included patient demographics (age, sex, race, body mass index (BMI), alcohol use), medical characteristics including hypertension, dyslipidemia, lung disease, peripheral artery disease, prior heart failure, prior myocardial infarction, chronic kidney disease (estimated glomerular filtration rate (eGFR) < 60 mL/min), glycated hemoglobin (HbA1C), and mitral valve disease/stenosis. We also collected preoperative and postoperative medication use including angiotensin-converting enzyme (ACE), angiotensin receptor blockers (ARB), acetylsalicylic acid (ASA), other anti-platelet, beta blocker lipid-lowering agents, steroids, vitamins C and E, amiodarone, and magnesium sulfate. Lastly, we extracted cardiac characteristics data such as active myocardial infarction (STEMI), coronary artery bypass graft (CABG), valve surgery, cross-clamp time, explant position, intra-aortic balloon pump, surgery status, and resuscitation.

### Exposure of interest

The primary exposure of interest was continuous metformin use at a dose of 500 mg or higher in effect before cardiac surgery as captured through the prior-to-admission medication listing using Clinical Decision Intelligence System (CDIS) at the study institution.

### Study sample

All adult patients with diabetes age 18 years or older undergoing any major cardiac surgery at Geisinger Health System from 1/1/2009 to 11/30/2014. Types of cardiac valve surgeries present were mitral and aortic, but no transcatheter aortic valve replacements. Patients were excluded if they had a history of AF or any other major cardiac arrhythmia before cardiac surgery.

### Sample size justification

A power analysis was performed based on the assumption that the incidence of postoperative AF would be 22% for patients with metformin and 30% for patients without metformin. In the Maisel et al. study, the incidence trend of postoperative AF ranged from 10 to 65% depending on patient characteristics [3]. In the Mathew et al. [4] and Mariscalco et al. [5] studies, the AF rate was 30% for patients without metformin. The study had 80.5% power to detect an absolute 8% difference between the metformin and non-metformin groups.

### Study end points

The primary end point was the incidence of postoperative AF. The event of AF was assessed by the recording of a 12 lead electrocardiogram, from the continuous bedside monitor characterized by the absence of discrete P waves and an irregularly irregular ventricular rate. The occurrence of any new atrial fibrillation during the post-operative period, (i.e., immediately after the planned cardiac surgery until discharge from the hospital for that admission) was identified as post-operative atrial fibrillation. We also examined 30-day hospital readmission rate due to any arrhythmia and in-hospital postoperative acute renal failure (defined as a rise in serum creatinine of more than three-fold from baseline).

### Propensity score matching

**Fig. 1:**
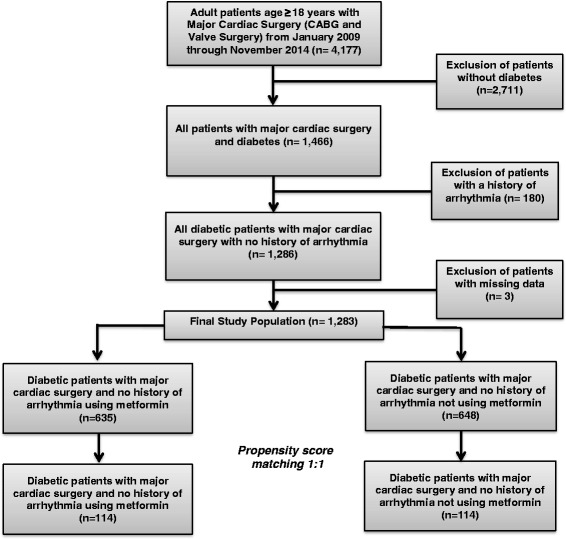
Propensity score matching flow diagram. Major cardiac surgery = CABG (coronary artery bypass graft) and cardiac valve surgery

The propensity score matching method was used to reduce selection bias introduced by using a non-randomized design (Fig. 1.) [19]. The following variables, chosen from the STS database, were used to compute the propensity score for each patient: age, sex, race, BMI, alcohol consumption, hypertension, dyslipidemia, lung disease, peripheral artery disease, prior heart failure, prior myocardial infarction, chronic kidney disease (eGFR < 60 mL/min), HbA1C, mitral valve disease/stenosis, ACE or ARB use, ASA use, other anti-platelet use, beta blocker use, lipid-lowering agent use, steroid use, vitamins C and E use, amiodarone use, magnesium sulfate use, active myocardial infarction, CABG, valve surgery, cross-clamp time, explant position, intra-aortic balloon pump, surgery status and resuscitation. The propensity score was estimated using logistic regression treating metformin use as the dependent variable. We then matched metformin users to non-users in a 1:1 ratio using the greedy method [20, 21]. Matching was evaluated using standardized differences [19–22]. If any standardized difference exceeded 10%, then the propensity model was re-evaluated and updated. The process was repeated until all standardized differences of the baseline variables were < 10%.

### Statistical analysis

Descriptive statistics were reported for the full sample and were stratified by the patients discharged with and without metformin therapy after cardiac surgery. Comparison between the groups before matching was accomplished using the chi-square or Fisher’s exact tests, and two-sample *t* test or Wilcoxon rank sum test, as appropriate. Comparison between the groups after matching was accomplished using the McNemar test or paired *t* test. We defined incident AF, arrhythmia, and acute renal failure as no AF, arrhythmia or acute renal failure being present 30 days before surgery (washout period = 30 days). A *p* value of less than 0.05 was considered statistically significant. Statistical analysis was performed using SAS version 9.4 (SAS Institute, Cary, NC).

## Results

After excluding patients without diabetes, those with a history of arrhythmia and patients with missing data, the final sample size consisted of 1283 patients. There were 635 patients in the metformin prior-to-admission group and 648 in the non-metformin group. Demographic information, medical characteristics, preoperative medications use, postoperative medications use, and cardiac characteristics are summarized in Table 1, stratified by patients with or without metformin use before admission.

**Table 1:** Comparability of the groups before matching

Variable	Metformin prior to admission (*n* = 635)	Non-metformin prior to admission (*n* = 648)	*p* value
Demographics			
Age, years, mean (SD)	65.1 (9.8)	66.6 (10.1)	0.0064
Sex (male)	448 (70.6%)	423 (65.3%)	0.0431
Race (white)	628 (98.9%)	633 (97.7%)	0.0944
BMI, kg/m^2^, mean (SD)	32.37 (6.0)	32.13 (6.9)	0.4981
Alcohol use ≥ 2 drinks per week	36 (8.8%) *N* = 409	27 (6.8%) *N* = 397	0.2900
Medical characteristics			
Hypertension	571 (89.9%)	599 (92.4%)	0.1117
Dyslipidemia	564 (89.0%)	572 (88.3%)	0.6985
Lung disease	133 (20.94%)	154 (23.8%)	0.2255
Peripheral artery disease	67 (10.6%)	78 (12.1%)	0.4007
Prior heart failure	28 (7.6%) *N* = 368	48 (13.6%) *N* = 352	0.0085
Prior MI	195 (33.1%) *N* = 590	234 (39.1%) *N* = 599	0.0308
CKD (eGFR < 60 mL/min)	243 (38.3%)	371 (57.3%)	< 0.0001
HbA1C ≥ 9%	104 (18.4%) *N* = 566	79 (13.0%) *N* = 607	0.0115
Mitral valve disease/stenosis	142 (22.5%)	169 (26.4%)	0.1127
Preoperative medications use			
ACE or ARB (within 48 h)	193 (47.0%) *N* = 411	155 (38.9%) *N* = 399	0.0197
ASA (within 5 days)	555 (87.4%)	558 (86.1%)	0.4955
Other anti-platelets	62 (9.8%)	58 (9.0%)	0.6170
Beta blocker (within 24 h)	533 (83.9%)	561 (86.6%)	0.1827
Lipid lowering agents (within 24 h)	572 (90.1%)	568 (87.7%)	0.1677
Steroids (within 24 h)	19 (3.0%)	25 (3.9%)	0.3942
Vitamin C	117 (18.4%)	108 (16.7%)	0.4076
Vitamin E	19 (3.0%)	16 (2.5%)	0.5653
Amiodarone	47 (7.4%)	52 (8.0%)	0.6758
Magnesium sulfate (inpatient)	27 (4.3%)	21 (3.2%)	0.3399
Postoperative Medications Use			
ACE or ARB	254 (41.1%)	252 (39.8%)	0.6258
ASA	593 (96.0%)	609 (96.1%)	0.9265
Other anti-platelets	110 (17.8%)	171 (27.0%)	0.0001
Beta blocker	583 (94.3%)	583 (92.0%)	0.0959
Cardiac characteristics			
Active MI (STEMI)	15 (2.4%)	16 (2.5%)	0.9008
CABG	492 (77.5%)	506 (78.1%)	0.7940
Valve surgery	227(35.8%)	243 (37.5%)	0.5149
Cross clamp time ≥ 120	72 (18.7%) *N* = 386	101 (24.4%) *N* = 414	0.0486
Explant position, aortic	1 (0.2%)	2 (0.3%)	0.5751
Intra-aortic balloon pump	77 (12.1%)	95 (14.7%)	0.1828
Surgery status			0.2790
Emergent	14 (2.2%)	20 (3.1%)	
Urgent	257 (40.5%)	238 (36.7%)	
Elective	364 (57.3%)	390 (60.2%)	
Resuscitation	4 (0.7%) *N* = 590	7 (1.2%) *N* = 599	0.3769

Patients in the metformin group, when compared to the non-metformin group, were more likely to be male (70.6 vs. 65.3%; *p* = 0.0431) and younger (65.1 vs. 66.6; *p* = 0.0064), respectively. With regard to medical characteristics, patients in the metformin group, when compared to the non-metformin group had lower prior heart failure (7.6 vs. 13.6%; *p* = 0.0085) and MI rates (33.1 vs. 39.1%: *p* = 0.0308), lower CKD rates (38.3 vs. 57.3%; *p* < 0.0001) and higher rates of HbA1C ≥ 9% (18.4 vs. 13.0%; *p* = 0.0197), respectively. Patients in the metformin group, when compared to the non-metformin group, were more likely to use ACE inhibitors or ARBs preoperatively within 48 h (47.0 vs. 38.9%; *p* = 0.0197) and less likely to use other anti-platelets postoperatively (17.8 vs. 27.0%; *p* = 0.0001), respectively. With regard to cardiac characteristics, patients in the metformin group also had lower rates of cross-clamp time ≥ 120 (18.7% vs. 24.4; *p* = 0.0486). All variables presented in Table 1 were used to conduct propensity score matching. After matching, no significant differences were observed for any of the variables between the metformin and non-metformin groups (see Table 2).

**Table 2:** Comparability of the groups after matching

Variable	Metformin (*n* = 114)	Non-metformin (*n* = 114)	Standardized differences
Demographics			
Age, years (SD)	66.0 (9.2)	66.0 (9.7)	0
Sex (male)	83 (72.8%)	84 (73.7%)	− 0.02
Race (white)	113 (99.1%)	114 (100.0%)	− 0.13
BMI, kg/m^2^ (SD)	32.73 (6.8)	32.3 (6.3)	0.07
Alcohol use ≥ 2 drinks per week	7 (6.1%)	4 (3.5%)	0.12
Medical Characteristics			
Hypertension	105 (92.1%)	105 (92.1%)	0
Dyslipidemia	107 (93.9%)	101 (88.6%)	0.19
Lung disease	29 (25.4%)	32 (28.1%)	− 0.06
Peripheral artery disease	11 (9.7%)	11 (9.7%)	0
Prior heart failure	12 (10.5%)	10 (8.8%)	0.06
Prior MI	40 (35.1%)	35 (30.7%)	0.09
CKD (eGFR < 60 mL/min)	49 (43.0%)	52 (45.6%)	− 0.05
HbA1C ≥ 9%	16 (14.0%)	17 (14.9%)	− 0.02
Mitral valve disease/stenosis	55 (48.3%)	51(44.7%)	0.07
Preoperative medications use			
ACE or ARB (within 48 h)	55 (48.3%)	54 (47.4%)	0.02
ASA (within 5 days)	98 (89.0%)	101 (88.6%)	0.01
Other anti-platelets	5 (4.4%)	9 (7.9%)	− 0.14
Lipid lowering agents (within 24 h)	103 (90.4%)	101 (88.6%)	
Steroids (within 24 h)	1 (0.9%)	2 (1.8%)	0.06
Vitamin C	21 (18.4%)	21 (18.4%)	0
Vitamin E	2 (1.8%)	1(0.9%)	0.08
Amiodarone	5 (4.4%)	7 (6.1%)	− 0.07
Magnesium sulfate (inpatient)	3 (2.6%)	3 (2.6%)	0
Postoperative medications use			
ACE or ARB	60 (52.6%)	52 (45.6%)	0.14
ASA	110 (96.5%)	110 (96.5%)	0
Other anti-platelets	23 (20.2%)	23 (20.2%)	0
Beta blocker	104 (91.2%)	106 (93.0%)	− 0.07
Cardiac characteristics			
Active MI (STEMI)	1 (0.9%)	1 (0.9%)	0
CABG	82 (71.9%)	82 (71.9%)	0
Valve surgery	53 (46.5%)	54 (47.4%)	− 0.02
Cross clamp time ≥ 120	19 (16.7%)	13 (11.4%)	0.15
Explant position, aortic	1 (0.9%)	1 (0.9%)	0
Intra-aortic balloon pump	12 (10.5%)	12 (10.5%)	0
Surgery status			
Emergent	2 (1.8%)	2 (1.8%)	0
Urgent	35 (30.7%)	32 (28.1%)	0.06
Elective	77 (67.5%)	80 (70.2%)	− 0.06
Resuscitation	1 (0.9%)	1 (0.9%)	0

Table 3 presents comparisons between metformin and non-metformin groups for primary and secondary outcome variables before matching. Results for overall (CABG or valve surgery) showed that 23.5% of patients in the metformin group had developed new AF, 0.5% had acute renal failure, and 0.6% had 30-day readmission with arrhythmia. In the non-metformin group, 26.5% patients developed new AF, 0.8% had acute renal failure, and 0.5% had 30-day readmission with arrhythmia. All comparisons between the metformin and non-metformin groups for the primary and secondary variables were not significant. Similarly, when comparing the two groups within CABG only and valve surgery only, there were no significant differences for new AF, acute renal failure, and 30-day readmission with arrhythmia.

**Table 3:** Analyses of primary and secondary outcome variables before propensity score matching

Variable	Metformin (*n* = 635) 49.5%	Non-metformin (*n* = 648) 50.4%	*p* value
Overall (CABG or valve surgery)			
New atrial fibrillation	149 (23.5%)	172 (26.5%)	0.2088
Acute renal failure This is defined as 3-fold increase in creatinine from baseline on admission)	3 (0.5%)	5 (0.8%)	0.7258
30-day readmission with arrhythmia	4 (0.6%)	3 (0.5%)	0.7232
CABG only	*N* = 408	*N* = 405	
New atrial fibrillation	81 (19.9%)	91 (22.5%)	0.3707
Acute renal failure	0 (0.0%)	2 (0.5%)	0.2479
30-day readmission with arrhythmia	2 (0.5%)	2 (0.5%)	0.9999
Valve surgery only	*N* = 143	*N* = 142	
New atrial fibrillation	38 (26.6%)	39 (27.5%)	0.8655
Acute renal failure	2 (1.4%)	0 (0.0%)	0.4982
30-day readmission with arrhythmia	1 (0.7%)	0 (0.0%)	0.9999

After matching, 26.3% of patients in the metformin group had developed new AF, 0.9% had acute renal failure, and none had 30-day readmission with arrhythmia while 30.7% of patients in the non-metformin group developed new AF, none had acute renal failure, and 0.9% had 30-day readmission with arrhythmia (Table 4). There were no statistically significant differences between the two groups on the primary and secondary outcomes. Similarly, when analyzing CABG and valve surgery separately, there were no differences in all outcomes between patients in the metformin and non-metformin groups after matching.

**Table 4:** Analyses of primary and secondary outcome variables after propensity score matching

Variable	Metformin (*n* = 114) %	Non-metformin (*n* = 114) %	*p* value
Overall (CABG or valve surgery)			
New atrial fibrillation	30 (26.3%)	35 (30.7%)	0.4658
Acute renal failure	1 (0.9%)	0 (0.0%)	NA
30-day readmission with arrhythmia	0 (0.0%)	1 (0.9%)	NA
CABG only	*N* = 85	*N* = 85	
New atrial fibrillation	18 (21.2%)	24 (28.4%)	0.2733
Acute renal failure	1 (1.2%)	0 (0.0%)	NA
30-day readmission with arrhythmia	0 (0.0%)	1 (1.2%)	NA
Valve surgery only	*N* = 57	*N* = 57	
New atrial fibrillation	19 (33.3%)	22 (38.6%)	0.5775
Acute renal failure	0 (0.0%)	0 (0.0%)	NA
30-day readmission with arrhythmia	0 (0.0%)	0 (0.0%)	NA

## Discussion

The main objective of our study was to investigate the role of metformin therapy in the prevention of postoperative AF in patients with diabetes who underwent cardiac surgery. The major findings of this propensity score matched study show that metformin use was not associated with a decreased risk of developing postoperative AF. The findings were similar even after separating the cardiac surgery into CABG or isolated valve surgery. Moreover, metformin use was also not associated with acute renal failure or 30-day readmission due to arrhythmia.

Several studies have suggested an important role of inflammation and oxidative stress as inciting factors in the development of postoperative AF in diabetic cardiac surgery patients [23–26]. A recent meta-analysis concluded that increased circulation inflammatory factors are associated with greater AF risk in the general population as well as for patients who underwent CABG [27]. Pre-operative use of statin drugs has shown promising results in preventing postoperative AF in multiple studies [28–30]. Some other novel therapies being tested for the prevention of this condition with conflicting beneficial effects are fish oil, vitamin C, vitamin E, polyunsaturated fatty acids, and corticosteroids [31–36]. Although metformin exerts an anti-inflammatory effect by reducing CRP levels [11, 12] in our study, it was not associated with lower postoperative AF risk in cardiac surgery patients.

Chang et al. [14] who performed a population-based study that included 645,710 patients with T2D found that metformin protected patients from developing new AF for at least 2 years during their 13-year follow-up period. However, this study did not include cardiac surgery patients. Moreover, in their study, the researchers only adjusted for 14 covariates, which did not include HbA1C, antioxidant use, and BMI. In our propensity score matched study, we controlled for 35 potentially confounding variables to better isolate the role of metformin in the development of postoperative AF in cardiac surgery patients.

One of the limitations of our study is that we did not have data on the exact use period of metformin. We abstracted metformin use data (a dose of 500 mg or higher in effect before cardiac surgery) from the prior-to-admission medication listing using CDIS at the study institution. The absolute bioavailability of oral metformin ranges from 40 to 60%, and absorption is complete within 6 h [37]; thus, patients receiving metformin in our study would be achieving steady-state plasma levels. However, it may be that the reduced inflammation requires a longer metformin use period. Previous research in clinical populations such as Cameron et al. found that metformin reduced inflammation with use period ranging from 4 to 6 months or longer [38]. In their retrospective cohort study, the use period of metformin was for at least 6 months, and in their randomized placebo-controlled study patients in the metformin arm received the medication for 4 months. Propensity score matching decreases selection bias by balancing measured covariates; the larger the number of covariates used to construct the propensity score, the better the balance between the groups. In our study, we used 35 variables to construct the propensity score which included many of the variables shown in the literature to have an effect on postoperative AF. Unfortunately, we did not have data regarding diabetes duration and could not adjust for this variable. Moreover, only randomization can balance the unmeasured confounders; thus, hidden bias may remain in the present study.

Another limitation in our study is that the vast majority of patients were white. The relationship between metformin use and postoperative AF may be different for other races/ethnicities. For example, African American individuals may have a significantly improved glycemic response to metformin when compared with white Americans [39]. Moreover, the Diabetes Prevention Program study, comparing Asian with white patients, showed greater risk reduction for T2D incidence from metformin use (38 vs. 24%) [40].

## Conclusions

In our study, prior use of metformin therapy was not associated with the incidence of postoperative AF in diabetic patients undergoing cardiac surgery. With only a few studies exploring the relationship between metformin use and postoperative AF conducted to date, future randomized controlled trials are needed with more diverse patient populations.

## Abbreviations

ACE: Angiotensin-converting enzyme
AF: Atrial fibrillation
ARB: Angiotensin receptor blockers
BMI: Body mass index
CABG: Coronary artery bypass graft
CDIS: Clinical decision intelligence system
CRP: C-reactive protein
IL-6: Interleukin-6
IQR: Interquartile range
STEMI: ST-segment elevation myocardial infarction
STS: Society of Thoracic Surgeons
T2D: Type 2 diabetes
